# α-Tocomonoenol Is Bioavailable in Mice and May Partly Be Regulated by the Function of the Hepatic α-Tocopherol Transfer Protein

**DOI:** 10.3390/molecules25204803

**Published:** 2020-10-19

**Authors:** Andrea Irías-Mata, Nadine Sus, Maria-Lena Hug, Marco Müller, Walter Vetter, Jan Frank

**Affiliations:** 1 Department of Food Biofunctionality, Institute of Nutritional Sciences, University of Hohenheim, Garbenstr. 28, D-70599 Stuttgart, Germany; andrea.iriasmata@ucr.ac.cr (A.I.-M.); nadine.sus@nutres.de (N.S.); maria-lena.hug@gmx.de (M.-L.H.); 2 Institute of Food Chemistry, University of Hohenheim, D-70599 Stuttgart, Germany; marcomueller17@t-online.de (M.M.); walter.vetter@uni-hohenheim.de (W.V.)

**Keywords:** α-tocopherol transfer protein (TTP) knockout mice, α-tocomonoenol, adipose tissue, blood, liver, depletion, vitamin E

## Abstract

Tocomonoenols are vitamin E derivatives present in foods with a single double bond at carbon 11’ in the sidechain. The α-tocopherol transfer protein (TTP) is required for the maintenance of normal α-tocopherol (αT) concentrations. Its role in the tissue distribution of α-11′-tocomonoenol (αT_1_) is unknown. We investigated the tissue distribution of αT_1_ and αT in wild-type (TTP^+/+^) and TTP knockout (TTP^−/−^) mice fed diets with either αT or αT_1_ for two weeks. αT_1_ was only found in blood, not tissues. αT concentrations in TTP^+/+^ mice were in the order of adipose tissue > brain > heart > spleen > lungs > kidneys > small intestine > liver. Loss of TTP function depleted αT in all tissues. αT_1_, contrary to αT, was still present in the blood of TTP^−/−^ mice (16% of αT_1_ in TTP^+/+^). Autoclaving and storage at room temperature reduced αT and αT_1_ in experimental diets. In conclusion, αT_1_ is bioavailable, reaches the blood in mice, and may not entirely depend on TTP function for secretion into the systemic circulation. However, due to instability of the test compounds in the experimental diets, further in vivo experiments are required to clarify the role of TTP in αT_1_ secretion. Future research should consider compound stability during autoclaving of rodent feed.

## 1. Introduction

**Figure 1:**
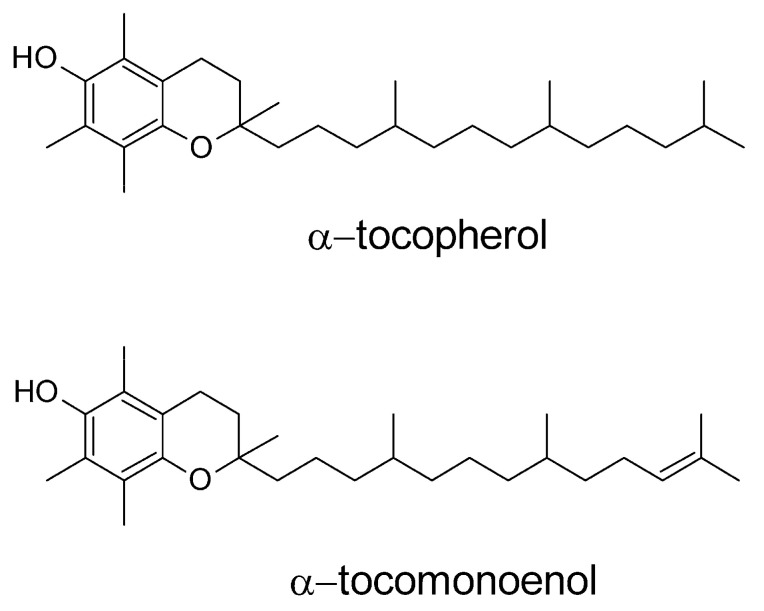
Chemical structures of α-tocopherol and α-11′-tocomonoenol.

The vitamin E family comprises eight structurally-related lipid-soluble compounds composed of a chromanol ring attached to a saturated (tocopherols (T)) or threefold unsaturated (tocotrienols) 16-carbon sidechain, with the prefixes α, β, γ, or δ designating the number and position of methyl groups substituted at the chromanol ring [1]. α-11′-Tocomonoenol (αT1; Figure 1), a novel vitamin E derivative with the structural feature of a single double bond at carbon 11’, was reported for the first time in 1995 [2] and later detected in palm, pumpkin, and sunflower oils [3,4,5].

From the gastrointestinal tract, all eight vitamin E congeners are absorbed and transported to the liver in a similar extent, but then the liver selectively secretes α-tocopherol (αT) into the bloodstream for distribution in the body, whereas the non-αT forms are preferentially metabolized via a cytochrome P_450_-dependent pathway [6]. It has been suggested that the selective retention of αT is the result of an interaction of the catabolic pathway with the hepatic α-tocopherol transfer protein (TTP) [7]. This is a cytosolic protein that preferentially binds αT (100%) over the other congeners, β-tocopherol (38%), γ-tocopherol (9%), δ-tocopherol (2%), and α-tocotrienol (12%) [8]. TTP is expressed primarily in the liver, but it has also been detected in other tissues, such as the rat brain, spleen, lung, kidney [9], rat uterus [10], and eye retina [11], suggesting that its expression in other organs regulates distinct tissue-specific accumulations of the vitamin [12].

Humans with mutations in the *Ttpa* gene encoding TTP develop ataxia with vitamin E deficiency (AVED) and are unable to maintain normal αT plasma concentrations [6,12]. Previous studies regarding the tissue distribution of αT alone or in combination with their homologues all-rac-αT, α-tocotrienol, γ-tocopherol, and γ-tocotrienol reported a wide distribution of αT in blood and tissues, primarily in the liver, lungs, spleen, and brain. The deletion of the *Ttpa* gene in mice (TTP knockout mice) leads to the depletion of αT from all tissues [7,13,14,15,16,17,18,19,20,21,22].

The uptake of dietary αT_1_ and its tissue concentration has only been studied in a single trial in mice, in which αT_1_ was detected in the liver and brain [23]. A deeper understanding of the tissue distribution, the accumulation of αT_1_, and their regulation (possibly by TTP) is required to elucidate its potential to exert the biological activity of vitamin E in mammals. We therefore investigated the tissue distribution of αT_1_ and αT in wild-type and TTP knockout mice (TTP^−/−^) following ingestion of the diet for two weeks.

## 2. Results and Discussion

### 2.1. Animal Performance

**Figure 2:**
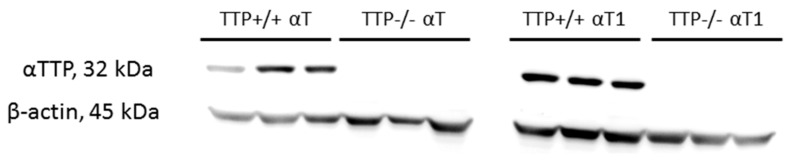
Representative Western blots of hepatic α-tocopherol transfer protein (TTP) expression in TTP+/+ and TTP−/− mice (n = 11) used in the feeding trial.

To investigate the tissue distribution of αT_1_ and its regulation by TTP, we fed 2–3-month-old wild-type (TTP^+/+^) and TTP knockout mice (TTP^−/−^, Figure 2) for two weeks with diets prepared with either 30 mg/kg αT_1_ or αT. No significant differences in body weight gain, final body weight, and absolute and relative (adjusted to body weight) organ weights were observed between the experimental groups (data not shown). This indicated a normal, healthy, and comparable performance of the mice during the experiment.

### 2.2. Bioavailability of αT and αT_1_ in TTP^+/+^ and TTP^−/−^ Mice

**Figure 3:**
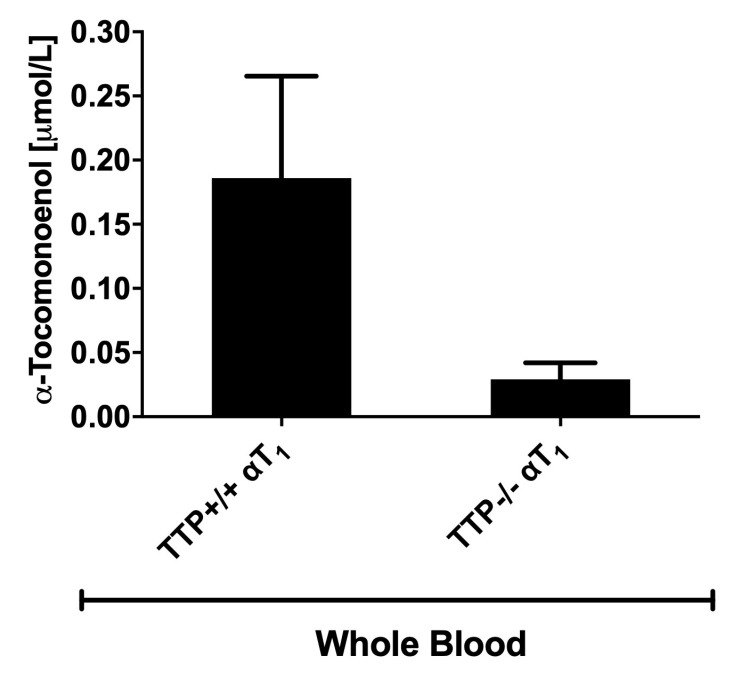
Mean whole blood concentration (error bars represent standard error of the mean; *n* = 11) of α-11′-tocomonoenol (αT_1_) in TTP^+/+^ and TTP^−/−^ mice fed a standard diet with either α-tocopherol (αT) or αT_1_ for 2 weeks.

To the best of our knowledge, this is the first time that the presence of αT_1_ is reported in the blood of TTP^+/+^ and TTP^−/−^ mice (Figure 3). In a previous mouse trial, αT_1_ was found in the liver and brain, but not in the blood [23]. Here, we found no αT_1_ in tissues, which is in partial agreement with the previous publication, where it was not found in the lung and spleen [23]. The absence of αT_1_ from tissues is likely explained by the low amounts of the compound ingested by our mice (see Section 2.3 for further details).

**Figure 4:**
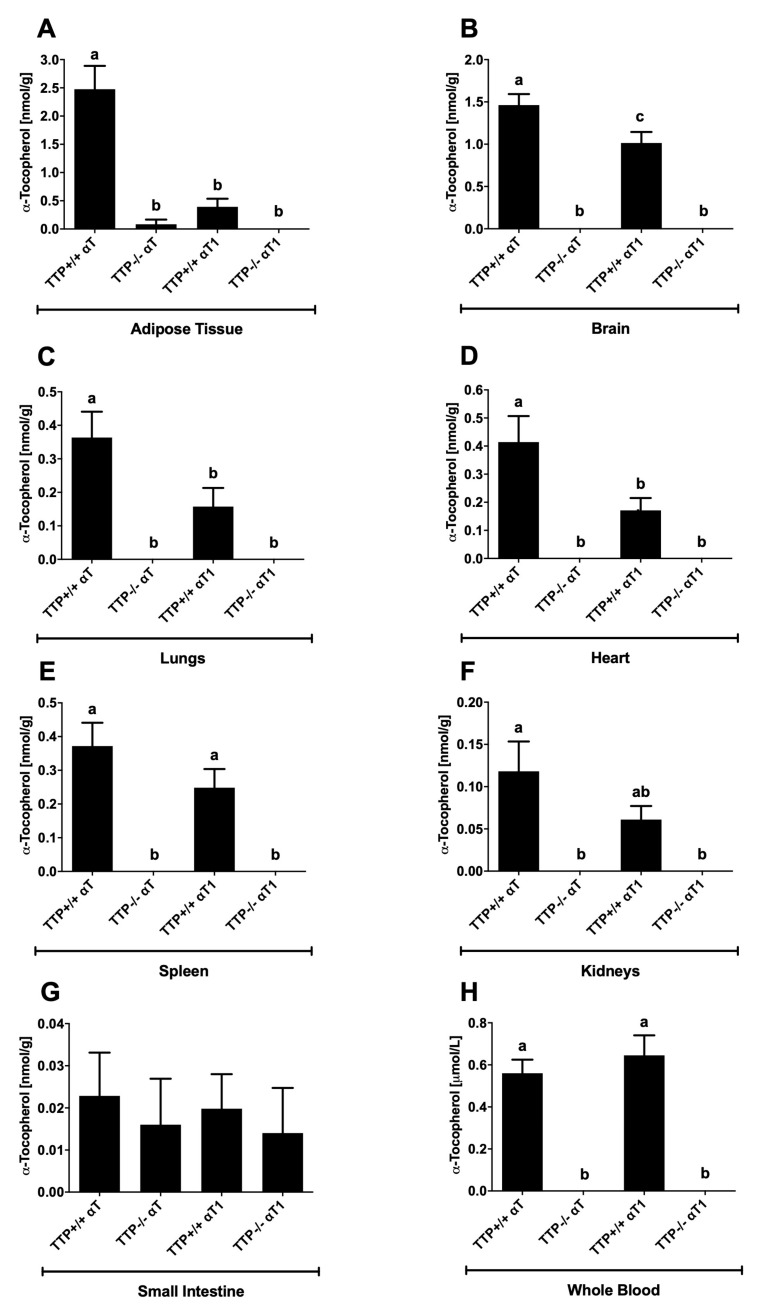
Mean tissue concentrations (error bars represent standard error of the mean; *n* = 11) of α-tocopherol (αT) in (**A**) adipose tissue, (**B**) brain, (**C**) lung, (**D**) heart, (**E**) spleen, (**F**) kidney, (**G**) small intestine, and (**H**) whole blood of TTP^+/+^ and TTP^−/−^ mice fed a standard diet with either αT or α-11′-tocomonoenol (αT_1_) for 2 weeks. Bars not sharing a common letter are significantly different at *p* < 0.05.

αT_1_ was numerically reduced in the blood of TTP^−/−^ mice, but contrary to αT (Figure 4), it was still present in the blood (Figure 3). Earlier publications reported a TTP-independent distribution of α-tocotrienol into the bloodstream [15]. Nevertheless, our results cannot rule out that TTP is involved in the distribution of αT1. The observed reduction in αT_1_ concentrations in the blood of TTP^−/−^ mice suggests that αT_1_ may not require TTP activity to the same extent as αT for secretion into the blood (Figure 4H), but there may still be a TTP influence on αT_1_ bioavailability. However, due to problems with the stability of αT_1_ in the experimental diets (see Section 2.3), these preliminary findings need to be interpreted with caution and require further substantiation by additional experiments.

αT was present in the blood and all examined tissues, with the exception of the liver, of TTP^+/+^ mice fed αT or αT_1_ (Figure 4). αT was also present in αT_1_-fed mice because all animals received αT-containing diets until the beginning of the trial, and the short duration of the trial did not result in a complete depletion of αT in the tissues. Another potentially contributing process that deserves further investigation is the conversion of αT_1_ to αT by sidechain saturation. Earlier investigations reported that the metabolism of tocotrienols involves enzymes, probably 2,4-dienoyl-coenzyme A-reductase, that catalyze the saturation of the sidechain, similar to those involved in the β-oxidation of unsaturated fatty acids [24]. This sidechain saturation would align with the observed concentrations of αT in the blood and tissues of TTP^+/+^ mice fed αT_1_.

αT concentrations in αT-fed TTP^+/+^ mice were in the order of adipose tissue > brain > heart > spleen > lungs > kidneys > small intestine > liver and reached 0.560 ± 0.065 μmol/L in blood (Figure 4). Relatively high αT concentrations in adipose tissue were reported before [13,16,25] and explained by the comparably low turnover of adipocytes, which slowly accumulated and released αT [16,26]. Adipose tissue was also reported as the main storage for α- and γ-tocotrienols and marine-derived tocopherol [14,16,18,25,27].

Overall, αT concentrations in tissues were about 10-fold lower than the values reported in tissues of mice and rats fed diets containing 30–100 mg αT per kg diet [7,13,16,18,25]. These low αT concentrations in tissues are more similar to values reported in mice and rats after feeding of αT-deficient or αT-free diets [16,25,28]. The unexpected depletion of αT and αT_1_ from the livers of our mice was confirmed by GC/MS analyses (see Figure S1, data interpretation based on a previous publication [29]). It was previously reported that αT is secreted from the liver at a higher rate, thus possibly depleting the liver of αT when the intake of the vitamin is inadequate [26,30]. A complete reduction of αT in the liver was reported for TTP^−/−^ mice fed a vitamin E-depleted diet from the age of 3 to 18 months [31], for male Wistar rats fed a diet containing < 0.4 μg/g αT for 6 weeks followed by an αT-free diet for 7 days [25], and an almost complete depletion of αT from the liver was observed in male Fisher 344 rats after 6 months feeding with an αT-deficient diet [32], as well as in male Wistar rats fed a vitamin E-free diet from 6 until 10 weeks old [16], but not in animals fed the recommended dietary dose of 30 mg/kg feed. The overall low αT and αT_1_ values in the tissues and their complete depletion from the liver led us to question the stability of the congeners in the experimental diets and the resulting amounts fed to our mice (see Section 2.3).

As expected, deletion of the *Ttpa* gene in mice resulted in almost complete depletion of αT from tissues and blood (Figure 4), highlighting its importance for the maintenance of an adequate αT status, as previously reported [7,13,22,29]. In agreement with our understanding of the role of TTP in mediating the selective secretion of αT from the liver into the bloodstream [6], αT concentrations in the small intestine were not under the control of TTP and therefore similar in TTP^+/+^ and TTP^−/−^ mice.

### 2.3. αT and αT_1_ in Experimental Diets

**Figure 5:**
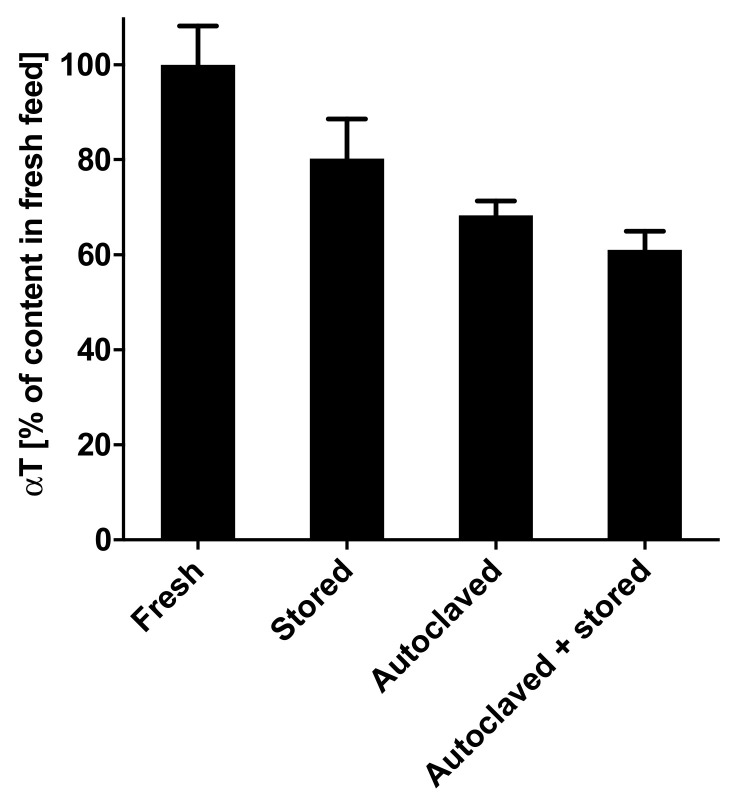
Mean total α-tocopherol (αT) content (error bars represent standard error of the mean; *n* = 3) of stored (1 week at room temperature with 12 h light/dark cycle), autoclaved (20 min at 121 °C), and autoclaved and stored rodent feed relative to fresh feed (not stored, not autoclaved, directly extracted and analysed). The C1000 rodent feed used was from Altromin Spezialfutter GmbH & Co (KG, Lage, Germany).

As mentioned above, the low αT concentrations in tissues and the depletion of αT and αT_1_ from the livers of our mice made us question the stability of both vitamin E congeners in the experimental diets fed to our animals. We therefore retrospectively measured the concentrations of αT and αT_1_ in the experimental diets, which were prepared with 30 mg of the respective congener per kilogram diet, but only found ≤1 mg/kg diet (Supplementary Figure S2). In the search of an explanation for this, we hypothesized that a sterilization procedure, such as autoclaving, which was performed on the diets prior to their transfer into the animal facilities, might partly explain the degradation. We therefore autoclaved a standard experimental diet with 30 mg/kg αT at 120 °C for 20 min and directly extracted for vitamin E analysis or stored them for one week at room temperature and a 12 h dark/light cycle before extraction and analysis. We also determined the vitamin E content in the diet before and after one week of storage at the conditions present in the animal facilities during the experiment. We observed a 32% reduction in αT after autoclaving, a 39% reduction after autoclaving and one week of storage, and a 20% reduction after one week of storage at the housing conditions of the mice (Figure 5). We therefore concluded that autoclaving and storage under housing conditions partly contributed to the degradation of vitamin E in the diets, but it does not entirely explain the massive losses of αT and αT_1_. It is also possible that the vitamin-E-free corn oil, in which αT and αT_1_ were diluted and then shipped to the manufacturer of the experimental diets for the preparation of our experimental diets, promoted the oxidation of the vitamin E congeners, and that the final concentrations of αT and αT_1_ in the diets were therefore significantly lower than planned.

Even though we currently cannot rule out alternative explanations, it is possible that the low intake of αT in our animals led to a redistribution of αT from the liver to peripheral tissues, resulting in the observed depletion of vitamin E from this organ and the lower values in the other tissues.

Notably, regardless of the low contents of αT and αT_1_ in the experimental diets, the presence of αT_1_ in blood is promising, as the bioavailability of αT_1_ is a requirement for further studies into any potential biological activities and health benefits of this dietary compound. The reported content of αT_1_ in food sources varies, but is generally low. Concentrations similar to those in our experimental diets, namely of 0.08–3.0 mg/kg [5] and below 10 mg/kg [4], have been reported for some varieties of palm oil and sunflower oil, respectively.

In conclusion, we report for the first time the absorption of αT_1_ into the blood of mice. Contrary to αT, αT_1_ was still found in the blood of mice not expressing TTP, but at lower concentrations than in wild-type mice. These results suggest that αT_1_ secretion from the liver may not, or not to the same extent as αT, depend on the function of TTP, but may still be partly controlled by it. However, additional experiments are required to substantiate or refute this.

We further conclude that significant losses of vitamin E in experimental diets are caused by autoclaving, which is frequently applied prior to the transfer of experimental feeds into specified pathogen-free animal facilities. Such losses need to be considered and concentrations of vitamin E in feeds should be analytically confirmed prior to using them in animal experiments.

## 3. Materials and Methods

### 3.1. Test Compounds and Diets

RRR-α-tocopherol (αT, ≥95%, CAS number 59-02-9, cat#KP5101) was from DSM (Grenzach, Germany), and α-11′-tocomonoenol (αT_1_; ≥99.5% pure) was extracted from vitamin E capsules as previously described [30]. αT and αT_1_ (165 mg each) were separately dissolved in 1 mL of ethanol and mixed thoroughly in 275 g vitamin E-stripped corn oil (Dyets, Bethlehem, PA, USA). The fortified oils were used in the preparation of semisynthetic standard rodent diets (vitamin E free standard diet, C1000; Altromin Spezialfutter, Lage, Germany) containing 5% oil and 30 mg/kg of either αT or αT_1_.

### 3.2. Animal Experiment

All animal procedures were carried out in accordance with the FELASA guidelines for the care and use of laboratory animals and approved by the Regional Council Stuttgart (Baden-Württemberg, Germany; trial no. V 342/17 BC). Forty-four female mice (2–3 months old, 21.2 ± 0.6 g) from our colony at the University of Hohenheim were fed for two weeks the αT-containing diet before 22 C57BL/6 wild-type mice (TTP^+/+^) and 22homozygous TTP knockout mice (TTP^−/−^, genotype confirmed by PCR) were randomized into four groups of 11 mice. Animals from each genotype were fed the experimental diets (modified C1000; Altromin) prepared with 30 mg/kg of either αT or αT_1_ for two weeks. Mice were housed in groups of maximum four per cage in a controlled environment (22 ± 2 °C, 55 ± 10% humidity, 12 h light/dark cycle) and had free access to food and water. After 2 weeks, animals were fasted for 12 h, anaesthetized with CO_2_, and killed by cervical dislocation. Blood was collected into heparinized tubes, and tissues (small intestine, liver, lungs, heart, kidneys, spleen, adipose tissue, and brain) were excised and snap-frozen in liquid nitrogen. All samples were immediately stored at −80 °C until further analysis.

### 3.3. HPLC Analysis

All chemicals used were of the highest purity and purchased from Sigma-Aldrich (Taufkirchen, Germany), JT Baker (Phillipsburg, NJ, USA), or Merck (Darmstadt, Germany). Methanol was HPLC- gradient grade and water was deionized and filtered (Millipore, Billerica, MA, USA). αT and αT_1_ were extracted from tissues (200 mg) and whole blood (100 µL) and saponified as previously described [33]. Prior to HPLC analysis, extracts were re-suspended in 100 μL methanol/water (85:15, *v/v*) and transferred to amber HPLC vials. Twenty microliters of the extract was injected into a Jasco HPLC (system controller LC-Net II/ADC, two pumps X-LC^TM^ 3185PU, mixing unit X-LC^TM^ 3180MX, degasser X-LC^TM^ 3080DG, autoinjector X-LC^TM^ 3159AS, column oven X-LC^TM^ 3067CO and fluorescence detector FP-2020 Plus; Jasco, Germany). Test compounds were separated on a Phenomenex Kinetex^TM^ PFP column (2.6 μm particle size, 150 × 4.6 mm) maintained at 40 °C using methanol/water (85:15, *v/v*) at a flow rate of 1.7 mL/min, for a total run time of 15 min. The fluorescence detector was operated at excitation/emission wavelengths of 296/325 nm, respectively. Peaks were recorded and integrated using Chrompass software (version 1.9. 302.1124, Jasco) and quantified against external standard curves using the authentic compounds.

### 3.4. GC/MS Analysis

To confirm the presence or absence of αT and αT1, crude extracts of liver samples were redissolved in 500 µL *n*-hexane, treated by column chromatography for purification, silylated, and analyzed by gas chromatography with mass spectrometry (GC/MS) as previously described [30].

### 3.5. Western Blot Analysis of TTP Expression

Liver protein homogenates were prepared in radioimmunoprecipitation assay buffer (Tris, 50 mM; NaCl, 150 mM; sodium dodecyl sulfate (SDS), 0.1%; sodium deoxycholate, 0.5%; Triton X100, 1%; EDTA, 20 mM (pH 7.2); dithiothreitol, 1 mM; protease inhibitor cocktail (Sigma-Aldrich)) and stored at −80 °C until further analyses. The amount of protein in the supernatant was determined by Bradford assay [34] and 40 μg of protein per lane was separated by 10% SDS gel electrophoresis and transferred to polyvinylidenefluoride membranes, blocked for 1 h at room temperature in blocking buffer (5% bovine serum albumin (Sigma-Aldrich) in Tris-buffered saline Tween-20 (TBST: 0.8% (*w/v*) NaCl, 0.24% (*w/v*) Tris-HCl (pH 7.6), 0.05% (*v/v*) Tween 20 in H_2_O_dd_; Sigma-Aldrich, Taufkirchen, Germany)), and incubated with the primary antibodies (TTP (1:1000, ab155323); β-actin (1:1000, #4967, Cell Signaling Technology, Danvers, USA)).

The primary antibodies were diluted in 5% bovine serum albumin in TBST and incubated overnight at 4 °C. Membranes were washed three times with TBST and incubated for 1 h at room temperature with the secondary antibody (goat anti-rabbit peroxidase conjugated (1:10,000, cat#401353, Calbiochem/Merck Millipore, Darmstadt, Germany)). Membranes were washed three times with TBST and bands were visualized using AceGlow^TM^ essential chemiluminescence solutions A and B (Peqlab Biotechnologie, Erlangen, Germany) and 20× LumiGLO^®^ reagent and peroxide solutions (Cell Signaling Technology, Cambridge, UK). Intensities were recorded on a Fusion FX imaging system, and band intensities were quantified using FusionCapt Advance software (Vilber Lourmat, Eberhardzell, Germany). Expression of the protein α-TTP was tested using the housekeeping protein β-actin as loading control.

### 3.6. Statistical Analysis

Statistical analyses were performed using GraphPad Prism 5 (GraphPad Software, San Diego, CA, USA). Differences between group means were calculated by one-way analysis of variance with a Tukey’s multiple comparison test or by unpaired *t*-test with Welch’s correction (heterogeneity of variances). Results are reported as means ± standard error of the mean (SEM). Differences were considered significant at *p* < 0.05.

## Supplementary Materials

The following are available online. Figure S1: Representative GC/MS chromatograms (full scan mode) of the analyzed liver extracts of TTP^+/+^ and TTP^-/-^ mice fed a standard diet with either α-tocopherol (αT; A and B, respectively) or α-11′-tocomonoenol (αT1; C and D, respectively) for 2 weeks. Figure S2: Mean concentrations of vitamin E in the diets used for breeding (A), standard (B) and experimental (C) diets fed the TTP^+/+^ and TTP^-/-^ mice during their life.
