# Supplementary material for: α-Tocomonoenol Is Bioavailable in Mice and May Partly Be Regulated by the Function of the Hepatic α-Tocopherol Transfer Protein
Source: Molecules. 2020 Oct 19;25(20):4803. doi: 10.3390/molecules25204803 (PMC7588010; doi:10.3390/molecules25204803)
Supplement: Supplementary file 1 [file molecules-25-04803-s001.pdf]

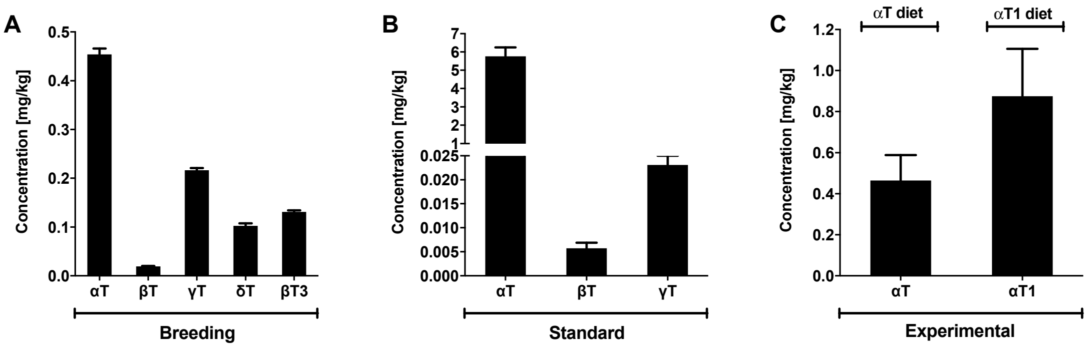

Supplementary Figure S1. Mean concentrations (error bars represent standard error of the mean; $n = 3$) of vitamin E in the diets used for breeding (A), standard (B), and experimental (C) foods fed the $TTP^{+/+}$ and $TTP^{-/-}$ mice during their lives. The standard food included all forms of vitamin E, and only two experimental foods were used: with α-tocopherol (αT) or with α-11′-tocomonoenol (αT_1). The breeding food was produced by ssniff Spezialdiaeten GmbH (Soest, Germany) and the standard and experimental diets were produced by Altromin Spezialfutter GmbH & Co. (KG, Lage, Germany).

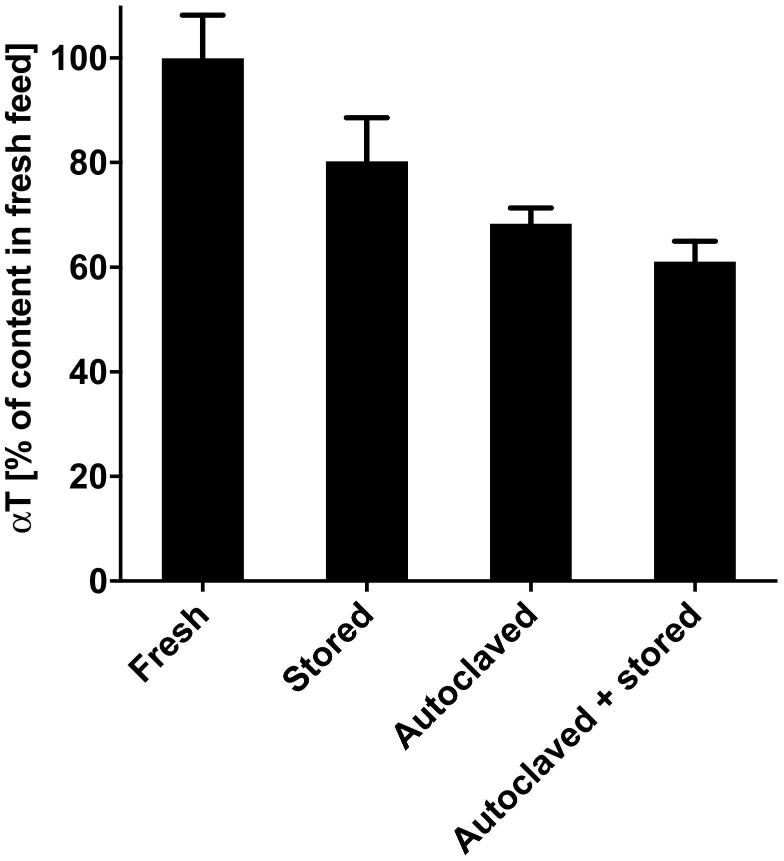

Supplementary Figure S2. Mean total α-tocopherol (αT) content (error bars represent standard error of the mean; n = 3) of stored (1 week at room temperature with 12 h light/dark cycle), autoclaved (20 min at 121 °C), and autoclaved and stored rodent feed relative to fresh feed (not stored, not autoclaved, directly extracted, and analyzed). The C1000 rodent feed used was obtained from Altromin Spezialfutter GmbH & Co. (KG, Lage, Germany).

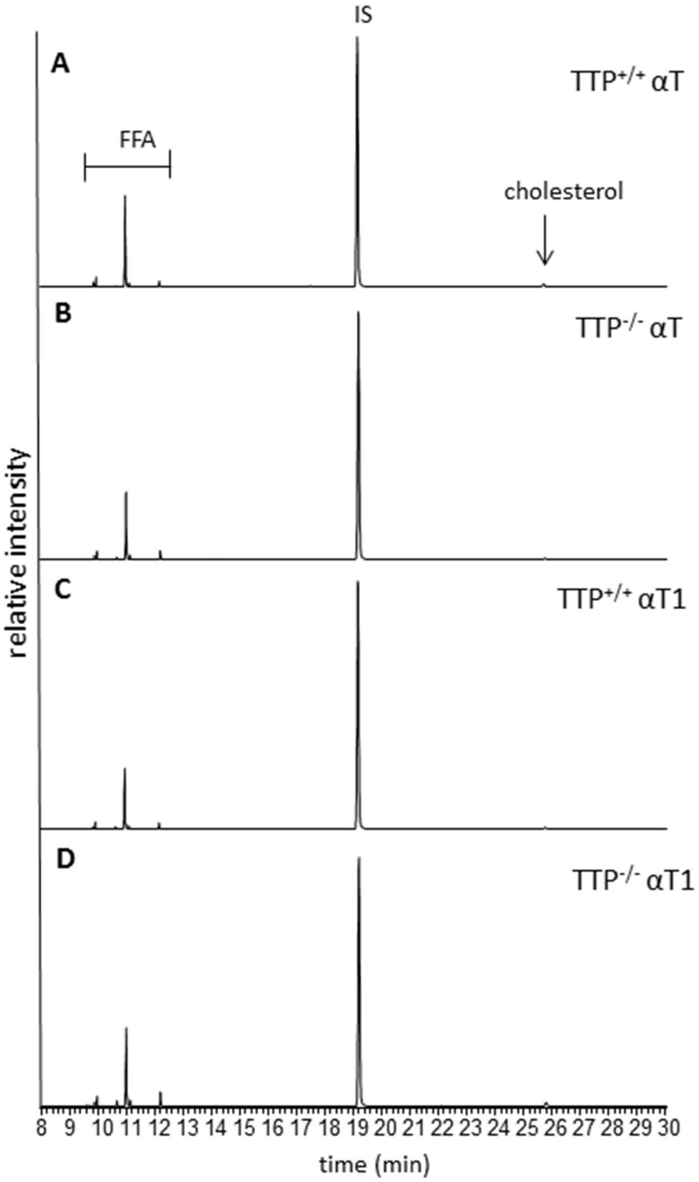

Supplementary Figure S3. Representative GC/MS chromatograms (full scan mode) of the analyzed liver extracts of $TTP^{+/+}$ and $TTP^{-/-}$ mice fed a standard diet with either α-tocopherol (αT; A and B, respectively) or α-11′-tocomonoenol (αT_1; C and D, respectively) for 2 weeks. Peaks from 10–12 minutes belong to free fatty acids (FFAs), the peak at 19 minutes is the internal standard (IS) 5-α-cholestane, and the slight peak at 26 minutes corresponds to cholesterol. αT and αT_1 were reported to appear at 24 and 25 minutes, respectively, and their absences were noted in all experimental groups.
